# Transglycosylation of Steviol Glycosides and Rebaudioside A: Synthesis Optimization, Structural Analysis and Sensory Profiles

**DOI:** 10.3390/foods9121753

**Published:** 2020-11-26

**Authors:** Ana Muñoz-Labrador, Silvana Azcarate, Rosa Lebrón-Aguilar, Jesús E. Quintanilla-López, Plácido Galindo-Iranzo, Sofia Kolida, Lisa Methven, Robert A. Rastall, F. Javier Moreno, Oswaldo Hernandez-Hernandez

**Affiliations:** 1 Institute of Food Science Research, CIAL (CSIC-UAM), Nicolás Cabrera 9, 28049 Madrid, Spain; ana.munoz@csic.es (A.M.-L.); o.hernandez@csic.es (O.H.-H.); 2 Consejo Nacional de Investigaciones Científicas y Técnicas (CONICET), Godoy Cruz 2290 CABA (C1425FQB), Argentina; silvanaazcarate@gmail.com; 3 Institute of Physical Chemistry ‘Rocasolano’ (IQFR-CSIC), Serrano 119, 28006 Madrid, Spain; rlebron@iqfr.csic.es (R.L.-A.); je.quintanilla@iqfr.csic.es (J.E.Q.-L.); pgalindo@iqfr.csic.es (P.G.-I.); 4 OptiBiotix Health PLC, Innovation Centre, Innovation Way, Heslington, York YO10 5DG, UK; skolida@optibiotix.com; 5 Department of Food and Nutritional Sciences, The University of Reading, PO Box 226, Whiteknights, Reading RG6 6AP, UK; l.methven@reading.ac.uk (L.M.); r.a.rastall@reading.ac.uk (R.A.R.)

**Keywords:** sweetener, CGTase, design of experiments, glycosylation, licorice, bitterness, steviol glycosides, rebaudioside A, *Stevia rebaudiana*

## Abstract

To improve flavor profiles, three cyclodextrin glucosyltransferases (CGTases) from different bacteriological sources, *Paenibacillus macerans*, *Geobacillus* sp. and *Thermoanaerobacter* sp., were used with an extract of steviol glycosides (SVglys) and rebaudioside A (RebA) as acceptor substrates in two parallel sets of reactions. A central composite experimental design was employed to maximize the concentration of glucosylated species synthesized, considering temperature, pH, time of reaction, enzymatic activity, maltodextrin concentration and SVglys/RebA concentration as experimental factors, together with their interactions. Liquid chromatography coupled to a diode-array detector (LC-DAD), liquid chromatography-mass spectrometry (LC-ESI-MS) and matrix-assisted laser desorption/ionization time-of-flight mass spectrometry (MALDI-TOF-MS) were used to characterize and identify the chemical structures obtained along the optimization. To assess the impact on the sensory properties, a sensory analysis was carried out with a group of panelists that evaluated up to 16 sensorial attributes. CGTase transglucosylation of the C-13 and/or C-19 led to the addition of up to 11 glucose units to the steviol aglycone, which meant the achievement of enhanced sensory profiles due to a diminution of bitterness and licorice appreciations. The outcome herein obtained supposes the development of new potential alternatives to replace free sugars with low-calorie sweeteners with added health benefits.

## 1. Introduction

The incidence of obesity is startling and estimated to increase to 38% of the world’s adult population by 2030 [1,2]. Member states of the World Health Organization (WHO) presented a voluntary target to stop the rise of obesity by 2025 [3]. The aim is to curb the increasing energy intake that has been a constant trend over the last decades, partially attributed to the overconsumption of added sugars [4,5]. In association with these trends, the prevention and treatment of obesity are of the utmost importance.

Evidence supporting reduced overall energy intake through the consumption of high-intensity sweeteners (HIS) in the place of free sugars has been reported [6,7]. The Joint FAO/WHO Expert Committee on Food Additives (JECFA) has established an acceptable daily intake (ADI) for the different HIS according to pharmacokinetic screening studies. Such compounds are represented by two types: HIS produced from non-natural sources, such as aspartame (ADI: 50 mg/kg/body weight (bw)/d), neotame (ADI: 0.3 mg/kg/bw/d), sucralose (5 mg/kg/bw/d), acesulfame K (ADI: 15 mg/kg/bw/d), saccharin (ADI: 15 mg/kg/bw/d) and cyclamate (11 mg/kg/bw/d); and HIS produced from natural sources, such as plant extracts including steviol glycosides (4 mg/kg/bw/d) and mogrosides (ADI not specified), among others [8]. Numerous artificial sweeteners have been claimed to be associated with a range of adverse effects due to their relationship with obesity, type 2 diabetes, neurology disorders and different types of cancer, unlike natural sweeteners, whose safety is extensively consensual [9].

Although many natural compounds are isolated for their sweetness, only a few have been developed for commercial use. The perennial herb plant *Stevia rebaudiana* (Bertoni) is a shrub of the family *Asteraceae*, native to Paraguay and Brazil. Stevioside and rebaudioside A are major constituents isolated from leaves and present a sweetness rate of 300–400 times greater than sucrose [10]. These compounds are used to sweeten food products and beverages as they have been regulated as food additives by the European Food Safety Authority (EFSA) [11] and approved as Generally Recognized as Safe (GRAS) natural sweeteners by the Food and Drug Administration (FDA) [12]. Health-promoting properties were described for most steviol glycosides, such as antioxidative, anti-inflammatory, immunomodulatory and anticancer properties. Toxicological studies have been carried out in mammalian species without having found significant adverse effects [13].

Steviol glycosides are formed on a common aglycone, steviol, and differ from each other only in the glycosidic constituents attached to C-13 and/or C-19. In addition, other steviol glycosides with a lower sweetness intensity are present as minor components, such as rebaudiosides B–F, rubusoside and dulcoside A. However, the use of steviol glycosides as a sweetener is compromised by the presence of unpleasant notes to the flavor profile [14,15].

Chemical and biochemical modifications have been developed to improve the sensory acceptance of these HIS. Cyclodextrin glycosyltransferase (CGTase, EC 2.4.1.19) is a key microbial amylolytic enzyme and a bacterial glycosyltransferase that catalyzes the conversion of starch and related polysaccharides into cyclodextrins (CDs). CDs are cyclic nonreducing oligosaccharides constituted by a variable number of glucose units linked by α1-4 glycosidic bonds. The most common forms are α-, β- and ɣ-CD depending on the number of glucose units, i.e., six, seven or eight, respectively. CGtases are known for their potential to improve the taste profile through transglycosylation activity [16,17,18].

CGTase-catalyzed glucosyl transfer reactions could synthesize a range of products with potentially useful applications. In this work, response surface methodology was used to study different variables and their interactions to assess the optimal conditions for glucosylation of steviol glycosides (Svglys) and rebaudioside A (RebA) by three different CGTases, consequently improving the sensory properties.

## 2. Materials and Methods

### 2.1. Materials

Steviol glycosides (59.4% w/w of stevioside and 25.4% w/w of rebaudioside A) and rebaudioside A (≥96% w/w) were purchased from Carbosynth (Berkshire, UK). CGTases from *Paenibacillus macerans*, *Geobacillus* sp. and *Thermoanaerobacter* sp. were employed. Stevioside, rebaudioside A, rebaudioside C and rubusoside standards were purchased from Biosynth Carbosynth (Reading, UK). White granulated sugar (Tate and Lyle, London, UK) and water (Harrogate Spa mineral water) for sensory analysis were purchased in local supermarkets in Reading (UK). All other reagents were obtained from Sigma-Aldrich (St Louis, MO, USA) and Thermo Fisher Scientific (San Jose, CA, USA).

### 2.2. Experimental Design of Transglucosylation of Steviol Glycosides

Optimization of the transglucosylation reactions with three different CGTase enzymes was carried out by response surface methods (Software Design Expert 10.1, StatEase, Minneapolis, MN, USA) to determine the significant factors out of all the parameters. The design involved screening the lowest and highest values of the chosen ranges [15] of the following experimental variables (Table 1): donor substrate concentration (maltodextrin from maize starch, 20 dextrose equivalents, 5–50 mg/mL), acceptor substrate concentration (Svglys/RebA, 5–50 mg/mL), enzyme activity (5–25 U/g Svglys/RebA), temperature (50–70 °C), reaction time (1–6 h) and pH (5–7). The resultant design consisted of 16 experiments to determine the optimal enzyme to proceed with the optimization. Once the optimum enzyme was chosen, the most influential parameters were studied using a fractional factorial design (2^(6-2)^) by representing the responses with the corresponding Pareto chart. The factors significantly affecting the response were then selected for a Central Composite Design (CCD) to obtain the optimal conditions for the glycosylation reactions. Accuracy and precision of the analytical assay method were evaluated using the relative percentage error (RE) and the appropriate relative standard deviation (RSD) from the theoretical concentrations of stevioside and rebaudioside A by external calibration.

### 2.3. Liquid Chromatography Coupled to a Diode-Array Detector (LC-DAD)

The concentration of the glucosylated products in the final reaction mixtures was determined by LC-DAD (Agilent 1200 series) equipped with an autosampler, quaternary pump, column oven and DAD detector. The separation was performed on a reversed-phase C18 column (Poroshell 120 C18 column; 250 mm × 4.6 mm, 4 μm particle size, 120 Å pore size; Agilent Technologies, Palo Alto, CA, USA) at a flow rate of 0.7 mL/min at 30 °C. The sample injection volume was 20 µL and the elution gradient using deionized water (eluent A) and acetonitrile (eluent B) varied from 90:10 (v:v) to 10:90 (v:v) in 50 min, then to initial conditions in 2 min and maintained for 10 min for conditioning.

### 2.4. Matrix-Assisted Laser Desorption/Ionization Time-of-Flight Mass Spectrometry (MALDI-TOF MS)

Molecular weight distributions of unmodified and glucosylated Svglys and RebA samples were determined by MALDI-TOF MS in a Voyager DE-PRO mass spectrometer (Applied Biosystems, Foster City, CA, USA), equipped with a delayed extraction ion source and a nitrogen laser emitting at 337 nm. An acceleration voltage of 25 kV, a 94% grid voltage, a 0.075% ion guide wire voltage and a delay time of 400 ns were applied. 2,5-Dihydroxybenzoic acid at a concentration of 10 mg/mL in water was used as a matrix. The different samples were diluted 100-fold with water and mixed with the matrix in an approximate ratio of 1:3 (v:v). One microliter of this mixture was spotted onto the flat stainless-steel sample plate and dried in air before analysis. All mass spectra were recorded over the *m/z* range 500–4000 in the linear positive ion mode, detecting glucosylated species as [M + Na]^+^. Average [M + H]^+^ values of the constituents of the Calibration Mixtures 1 and 2 (Sequazyme Peptide Mass Standards Kits, Applied Biosystems) were used for external mass calibration.

### 2.5. Liquid Chromatography-Mass Spectrometry (LC-MS)

An Agilent 1100 Series (Agilent Technologies) LC system (equipped with an autosampler, a quaternary pump and a column oven) coupled to an HTC-Ultra ETD II ion trap mass spectrometer (Bruker Daltonics, Fremont, CA, USA) by an electrospray (ESI) interface was used for LC-MS analyses. Data acquisition and processing were managed by Bruker Compass 1.2 software (Bruker Daltonics).

Unmodified and glucosylated Svglys and RebA samples were diluted 10-fold with water and separated on a C18 HyPURITY^TM^ column (100 mm × 2.1 mm, 3 µm particle size, Thermo Fisher Scientific, Waltham, MA, USA) at a flow rate of 0.2 mL/min at 35 °C. A binary gradient of water (eluent A) and acetonitrile (eluent B), both with 0.1% formic acid in water, was used. The elution program starts with 1 min in isocratic conditions (10% B). The percentage of B is then linearly increased in 44 min up to 50% and maintained for 5 min. Later, B increases from 50 to 90% B in 1 min holding for 9 min. Finally, the initial elution composition is reached in 1 min and maintained for 10 min to column equilibration. The injection volume was 5 µL.

Working conditions for the ESI source were: spray voltage, 3.5 kV; skimmer voltage, −40 V; nebulizer pressure (nitrogen), 40 psi; drying gas flow rate (nitrogen), 10 L/min; and drying gas temperature, 350 °C. Mass spectra were acquired in the negative mode, scanning from *m/z* 100 to 2000.

### 2.6. Isolation of Modified Steviol Glycosides and Rebaudioside A for Sensory Analysis

One hundred milliliters of reaction were placed into a Diaion HP-20 column (2.2 × 50 cm). The column was washed with 1500 mL of deionized water. Modified Svglys and RebA were eluted with ethanol (95%) and subsequently dried using a rotary evaporator (40 °C). Samples were kept at 4 °C until analysis.

### 2.7. Sensory Analysis

The sweetness intensity of the unmodified and glucosylated Svglys and RebA obtained in the optimal synthesis conditions was evaluated using 10 experienced sensory evaluation panelists. The panelists were trained at the Sensory Science Centre (Department of Food and Nutritional Sciences, University of Reading, UK). The study was approved by the University of Reading Research Ethics Committee (UREC Study Number: 16_19). Sensory analysis was performed in an air-conditioned (23–24 °C, room temperature) sensory laboratory with individual booths and artificial daylight. Sample order presentation was done in a balanced monadic sequential manner. All samples were rated in triplicate on separate days.

The panel used 16 attributes to describe the samples (sweet, overall strength of off-taste/flavor, bitter taste, licorice flavor, sour taste, cooked sugar flavor, cooling sensation, cardboard/stale, metallic, salty taste, crusty bread flavor, perfume flavor, sweet aftertaste, bitter aftertaste, licorice aftereffect and cooling aftertaste), followed by training focused on ensuring each panelist could reliably score sweetness relative to four sucrose standards (20, 30, 60 and 80 g/L). The average panel ratings for these standards were 10, 35, 75 and 100, respectively, on a 0–100 line scale, and these four positions were used as anchors to provide a structured scale on which to rate the sweetness of all samples. All other attributes were scored as relative values using unstructured line scales (0–100). Due to the limited sample availability, each panelist was presented with only 0.5 mL of sample for each scoring session. Therefore, training additionally focused on ensuring panelists were able to sip this small sample volume from a 30 mL transparent polystyrene cup and allow it to flow over the top of their tongue before swallowing and scoring sweetness accurately. Palate cleansing before and between sample scoring was done using filtered water and low salt crackers (Carr’s water crackers, United Biscuits Ltd., Hayes, UK).

Samples were prepared in mineral water (Harrogate Spa mineral water) and labeled with random 3-digit codes. Sample order presentation was done in a balanced monadic sequential manner. An initial screening was carried out to choose the most adequate concentrations for both the initial samples and the enzymatically modified. This was concluded as 0.32 g/L for Svglys and 0.24 g/L for the RebA samples.

The sucrose standards were presented at the start of each panel rating session for refamiliarization to enable the panelists to score the sweetness of the samples accurately against the standard anchors.

The mean sweetness ratings of the four sucrose standards were used to plot a dose-response curve, the linear regression for which was Perceived Sweetness = (1.55 × Sucrose Concentration (g/L)) − 22.5 (r^2^ = 0.99). The mean sweetness ratings for each sample were converted to equivalent sweetness (ES) values using this equation.

The sensory profile data were analyzed using a mixed-model ANOVA where panelists were treated as random effects and samples as fixed effects. The main effects were tested against the sample by assessor interaction. Multiple pairwise comparisons were carried out using Fisher’s LSD and a significant difference was declared at an alpha risk of 5% (*p* ≤ 0.05). Data analysis was carried out using the Senpaq software (Qi Statistics, Reading, UK).

## 3. Results

### 3.1. Optimization of Steviol Glycosides and Rebaudioside: A Transglucosylation Parameters by Response Surface Methodology

Initially, a screening using a fractional factorial design 2^(6-2)^ was carried out with three CGTases from *Paenibacillus macerans*, *Geobacillus* sp. and *Thermoanaerobacter* sp., respectively. The response variable was the concentration of transglucosylated products (mg/mL) quantified by LC-DAD. The values of the six experimental factors to be evaluated (concentration of maltodextrin, concentration of unmodified Svglys and RebA, enzymatic activity, temperature, time and pH), and the corresponding results for the three different enzymes are presented in Table 1. The average RE of stevioside and rebaudioside A were 5.4% (RSD = 4.2%, *n* = 4) and 4.6% (RSD = 4.4%, *n* = 4), respectively, suggesting that the quantification method could provide sufficient accuracy and precision for the quantification of the samples.

**Figure 1:**
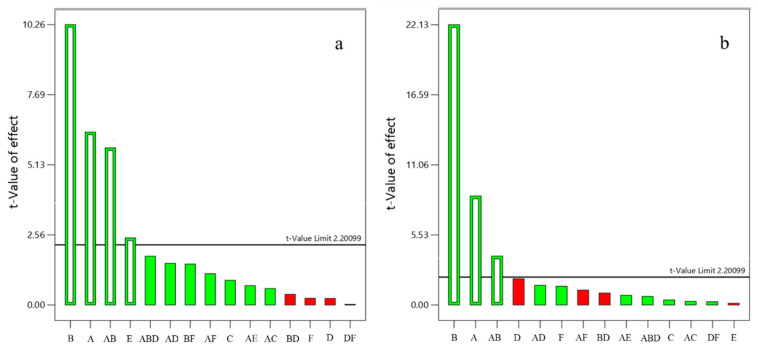
Pareto chart showing individual and interaction effects of the response evaluated: synthesized glucosylated steviol glycosides (SVglys) (**a**) and synthesized glucosylated rebaudioside A (RebA) (**b**) using cyclodextrin glucosyltransferases (CGTase) from *Geobacillus* sp. (A) Maltodextrin concentration, (B) initial SVglys/RebA concentration, (C) enzyme activity, (D) temperature, (E) time and (F) pH. Using a confidence value of *p* = 0.05, based on a null hypothesis test, values exceeding this limit (horizontal line) are considered significant to the response values.

The CGTase from *Geobacillus* sp. was selected to carry on the next steps of optimization, as this resulted in the highest concentration of glucosylated products for Svglys (11.33 mg/mL) and RebA (28.51 mg/mL). Thereafter, the factors affecting the response were evaluated through a Pareto chart (Figure 1) illustrating the analysis of variance (ANOVA) and *p*-value.

Positive values (green bars) denote a directly proportional relationship of the variable with the response, whereas negative values (red bars) reflect an inverse relationship. The horizontal line corresponds to the *t*-value at a significance level of 5%. The concentration of maltodextrin and Svglys and the time of reaction had a significant effect on the formation of glycosylated Svglys (Figure 1a), whereas only the concentration of maltodextrin and RebA had a significant effect on the formation of glycosylated RebA (Figure 1b).

A CCD optimization was performed to study the three significant factors obtained setting the resting parameters at the lowest level. The CCD design was composed of 17 runs for SVglys and 11 runs for RebA, with 3 replicates in the central point. An optimization phase was performed by applying response surface methodology (RSM) to optimize product formation. The relationship between the response evaluated and the variables for glucosylated SVglys (Equation (1)) and glucosylated RebA (Equation (2)) was fitted into the polynomial equations as follows:

Glucosylated SVglys (mg/mL) = −267.20 + 0.08 * maltodextrin (mg/mL) + 0.11 * unmodified SVglys (mg/mL) + 102.66 * time (h) (1)

Glucosylated RebA (mg/mL) = 0.17 + 0.08 * maltodextrin (mg/mL) + 0.38 * unmodified RebA (mg/mL) (2)

Analysis of variance (ANOVA) was carried out to determine the significance and adequacy of the fit of the regression model. Statistical significance of the model was established at *p* ≤ 0.05. The F-values of the obtained model (F < 0.03) for the response indicate that the mode was highly adequate and significant. Likewise, the determination coefficients (R^2^) of the model were 0.85 for glucosylated SVglys and 0.90 for RebA. Moreover, the coefficient of variation (CV %) was lower than 10%, showing that the variation was acceptable and satisfactory.

**Figure 2:**
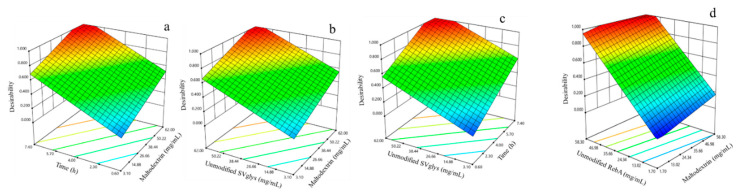
Three-dimensional plots showing the desirability of the maximization of synthesized glucosylated SVglys for the three significant variables (**a**–**c**) and synthesized glucosylated RebA for the two significant variables (**d**).

After that, the aim was to find the optimum concentration of unmodified SVglys and enzymatic activity to maximize the synthesized glucosylated SVglys and glucosylated RebA (mg/mL). The response surface obtained for the global desirability function (D) is presented in Figure 2. The coordinates producing the maximum desirability value (D = 1) for SVglys were 60.8 mg/mL for the concentration of maltodextrin, 59.7 mg/mL for the concentration of unmodified SVglys and 6.8 h for the time of reaction. For RebA, these were 51.9 mg/mL for the concentration of maltodextrin and 57.4 mg/mL for the concentration of unmodified RebA.

The individual response values and their respective confidence intervals are depicted in Table 2. To validate this predictive model, optimal conditions were experimentally assessed through three replicates and these showed no significant differences with the theoretical results. Finally, these conditions were selected to produce higher quantities of glucosylated SVglys and RebA to be structurally characterized and for the sensory analysis.

### 3.2. Structural Characterization by Mass Spectrometry

**Figure 3:**
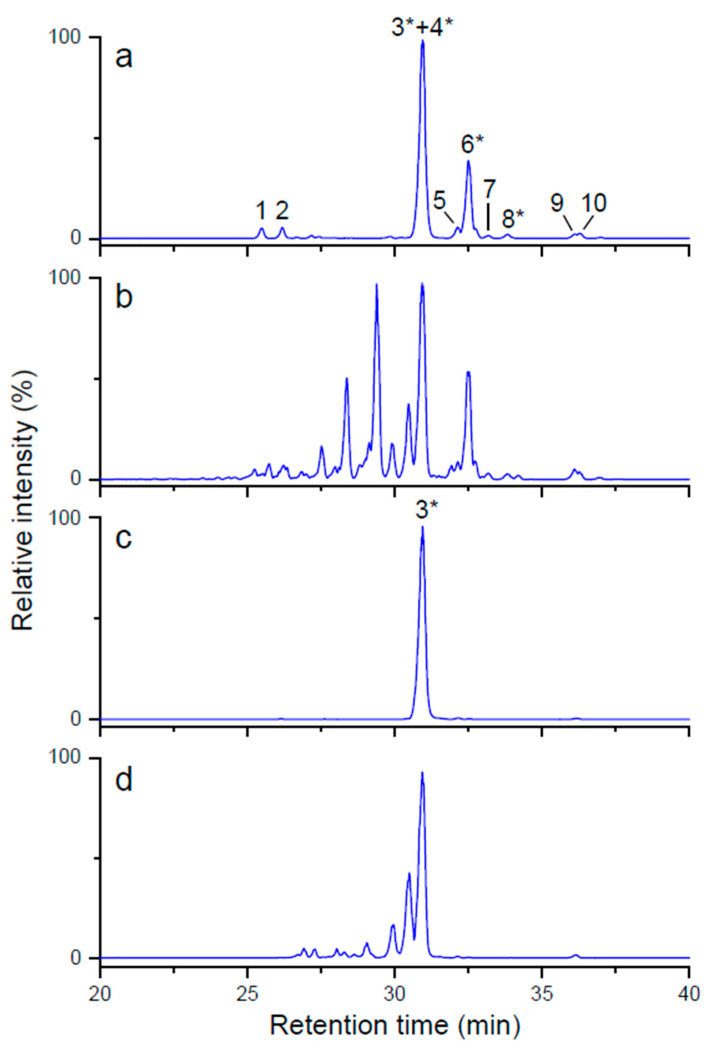
Base peak LC-MS chromatograms of unmodified SVglys (**a**), glucosylated SVglys (**b**), unmodified RebA (**c**) and glucosylated RebA (**d**) under optimized conditions using the CGTase from *Geobacillus* sp. Peak identification (peaks marked with asterisks were compared with the respective commercial standards): 1, 965.3 (unknown); 2, 1127.4 (rebaudioside D); 3, 965.3 (rebaudioside A); 4, 803.3 (stevioside); 5, 935.3 (rebaudioside F); 6, 949.6 (rebaudioside C); 7, 803.3 (rebaudioside G); 8, 641.3 (rubusoside); 9, 803.3 (rebaudioside B); 10, 641.3 (steviolbioside).

A comprehensive mass spectrometric approach using LC-ESI-MS and MALDI-TOF MS was carried out to reveal the structural modifications after the optimized reactions. CGTase from *Geobacillus* sp. generated the highest concentration of glycosylated structures for both SVglys and RebA as determined by LC-DAD (Table 1). LC-ESI-MS confirmed the results of LC-DAD and revealed similar profiles with the same retention times (Figure 3). Because the ionization mode was negative, most of the *m/z* data are [M - H]^−^ ions for the respective glycosides. Rebaudioside A and stevioside were not able to be separated successfully, whereas many of the glucosylated derivatives were totally or partially resolved. Some of the glycosides of the unmodified and optimal SVglys and RebA samples were identified by comparison with the retention time and the mass spectrum of commercial standards: rebaudioside A (*m/z* 965.3), stevioside (*m/z* 803.2), rebaudioside C (*m/z* 949.4) and rubusoside (*m/z* 641.3). Other glycosides were tentatively identified by relative retention and molecular masses reported in the literature [19,20]. Chromatographic peaks for higher *m/z* values as 1289.3 and 1451.3 were hypothetically considered as glucosylated glycosides up to seven glucoses in C-13 and/or C-19 positions.

**Figure 4:**
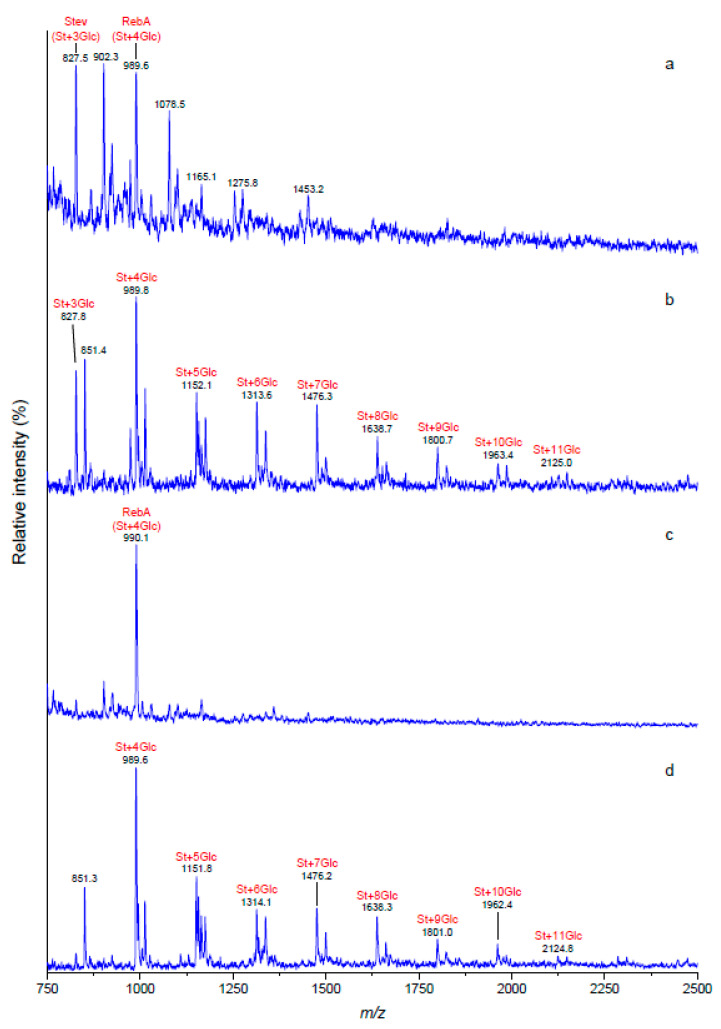
MALDI-TOF MS profile of unmodified SVglys (**a**), glucosylated SVglys (**b**), unmodified RebA (**c**) and glucosylated RebA (**d**) optimized conditions using the CGTase from *Geobacillus* sp. St: steviol; Stev: stevioside; RebA: rebaudioside A; Glc: glucose.

In accordance with the results obtained by LC-DAD and LC-ESI-MS, MALDI-TOF MS analysis showed the presence of glucosylated structures. However, MALDI-TOF spectra showed a series of [M + Na]^+^ ions, indicating extension glycoside chains for the three CGTases compared to the unmodified structures. As an example, Figure 4 shows the MALDI-TOF MS profiles of unmodified SVglys and RebA and their corresponding glucosylated forms catalyzed by the CGTase from *Geobacillus* sp.

Glucosylation of a mixture of steviol glycosides at both C-13 and C-19 sites by CGTase from *Bacillus subtilis* was previously described [21]. The LC-MS and MALDI-TOF MS data showed that the transglucosylation was equally efficient for RebA and SVglys (Figure S1). As shown in Figure 4b,d, SVglys and RebA contained up to 11 glucose residues, respectively, indicating successful tranglucosylation at the C-13 and/or C-19 sites of the steviol aglycone for both samples.

### 3.3. Sensory Profiling

Steviol glycosides such as stevioside and rebaudioside A were described to exhibit a bitter taste and licorice flavor characteristics. In this context, a sensorial analysis of the optimal samples obtained after the experimental design was carried out characterizing 16 flavor attributes using sucrose as reference.

**Figure 5:**
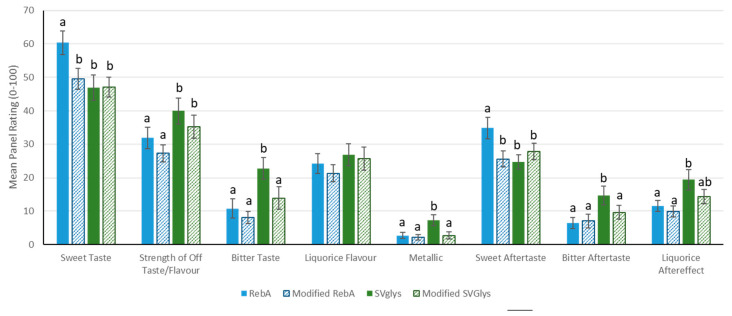
Mean panel ratings of attributes that either differed significantly between samples (different letters indicate *p* < 0.05 from Fisher’s LSD) or where mean rating > 10. Attributes that were not significantly different between samples or rated < 10 were: sour taste, salty taste, cooked sugar flavor, cooling sensation, stale flavor, crusty bread flavor, perfume and cooling aftereffect.

As shown in Figure 5, of the 16 attributes rated, 7 were significantly different between the samples. It was reported for natural steviol glycosides with β-D-glucopyranosyl units as constituents that the ratio of the glucose units at C-13 to C-19 of the steviol core has a relationship with the sweetness as well as with the quality of taste of the steviol glycosides [15]. The possible glucosidic linkages formed at C-19 of RebA could also have an impact on the bitter aftertaste; however, there was no significant effect on bitter taste by modification of the RebA in this. Additionally, the bitter taste was significantly and substantially higher for unmodified SVgly, mainly for consisting of a mixture of stevioside and rebaudioside A than for unmodified RebA, whose results were in accordance with previous reports, which stated that rebaudioside A is preferred for its sweetness and for being devoid of aftertaste bitterness over stevioside [22,23,24]. Likewise, regarding the sweetness, the initial RebA was significantly sweeter than the initial SVglys due to the quantity of stevioside present in this mixture, whose values are in agreement with the literature, where it is reported that rebaudioside A is 250–450 and stevioside 250–300 times greater than the sweetness of sucrose [25]. However, the sweetness of the RebA (Figure 5) was significantly reduced after the treatment with the CGTases compared to the unmodified RebA. The modified SVglys and modified RebA did not differ in sweetness (mean ratings of 47.1 and 49.6, respectively). However, sucrose equivalent (%) and the sweetness potency for SVglys and RebA and their corresponding modified samples, respectively, did not show significant changes (Table 3). All the other sensorial attributes measured were rated at low levels, except the licorice flavor, which did not differ between the RebA and SVglys samples, with or without modification. Importantly, a metallic taste, bitter taste and bitter aftertaste of SVglys were significantly reduced following their glucosylation by CGTase from *Geobacillus* sp. (Figure 5).

## 4. Discussion

Although stevioside and rebaudioside A are the most abundant of all steviol glycosides extracted from the leaves of the *Stevia rebaudiana* plant, its lingering bitterness limits applications as a sweetener in low-calorie foods and beverages formulations. Sweet taste reception is commenced by the activation of a heterogenic receptor, made up of a combination of hTAS1R2 and hTAS1R3 proteins, whereas the bitter taste in humans is mediated by 25 receptors of the hTAS2R gene family [20,23]. However, the specific mechanism of the flavor response is uncertain. A significant relationship between the structure and the sweetness of steviol glycosides has been described [15], and the flavor profile is determined by the glycosyl moieties with linkage- and regiospecificity. Ohta et al. [26] reported that the transglucosylation of both C-13 and C-19 of the steviol core is essential for sweetness. The number of glucose residues at C-13 seems to influence not only the intensity of sweetness but also the quality of taste (diminution of bitter aftertaste). This is evidenced by the lower bitterness of rebaudioside A compared to stevioside, which has only two glucose units at C-13, one less than rebaudioside A [25,27].

Cyclodextrin glucosyltransferases (CGTase) enzymes incubated with maltodextrin as a donor substrate were considered to be a promising tool for natural product glycosylations. The most common producers of CGTase are *Bacillus* sp. [17]. Many publications report the use of CGTase with steviol glycosides as acceptor substrates to improve the gustatory characteristics [15]. In the present work, the transglucosylation of a Stevia extract composed of a mixture of steviol glycosides (SVglys) and the single glycoside rebaudioside A (RebA) was studied. The mentioned transglucosylation reactions were carried out with three different sources of origin CGTases without delving into the literature with the specific acceptors herein utilized. The optimization considered different incubation conditions, which can greatly affect the hydrolysis and transglucosylation catalysis reactions of the CGTase enzymes. This work conducted an outstanding optimization through a comparative analysis of the yields obtained for the three CGTase enzymes by using a response surface approach (Table 1).

The reaction products were subjected to structural analysis using LC-DAD, LC-ESI-MS and MALDI-TOF-MS. Prakash et al. [21] carried out a maximum transglycosylation of up to eight glucose units for both SVglys and RebA samples using a CGTase produced by *Bacillus stearothermophilus*. Likewise, individual stevioside was also efficiently transglucosylated by using CGTases from different *Bacillus* sp. [28,29,30]. In all cases, the transglucosylation reactions yielded a mixture of mono- to over multiple-(α1-4)-glucosylated products, reflecting indiscriminate elongations of the C-13-β-sophorosyl unit and the C-19-ester-linked Glc(β1- residue, as reviewed by Gerwig et al. [15]. The optimal reactions in our work resulted in the addition of up to 11 glucose units for both modified SVglys and RebA reaching a higher glucosylation rate than those reported in the literature (Figure 4). By considering the well-described mechanisms of transglycosylation activity and substrate specificity of bacterial CGTases [31], as well as the high yield and efficiency in the transglucosylation reactions shown in our work, it is expectable that the transglucosylated products reported here are based on the exclusive transfer of (α1-4)-glucose residues to both the C-19-carboxyl group and C-13 hydroxyl group.

In addition, to investigate the impact of modification, a sensory analysis was carried out. The modification of SVglys did not reduce the sweetness and yet it significantly reduced the bitter taste, metallic taste and bitter aftertaste; however, the persistence of the licorice flavor note remained an issue. This behavior resembles the results obtained by Abelyan et al., who, despite the effectiveness of transglycosylation, did not completely remove the bitterness and residual aftertaste in stevioside modified with CGTases [30]. However, unlike the results herein obtained, Fukunaga et al. incubated stevioside with CGTase from *Bacillus macerans,* yielding decaglucosylated products, and yet the elongations that occurred led to an increase in bitterness [29]. Differently, the modified RebA sample experienced a significant reduction of its sweetness and sweet aftertaste and reduced attributes such as bitter licorice and metallic tastes and licorice aftereffects, although slightly. The detailed sensory study of the enzymatically modified samples revealed an optimal improvement on the set of flavors to be applied as sweeteners in food formulations. Taking into account both the sweetness properties and the potential obtained for SVglys and RebA, which are the main characteristics to its ability as a sweetener, they would still be considered HIS. These synthesized sweeteners would provide beneficial effects attributed to their natural counterpart, steviol glycosides, the genotoxicity of which have been extensively studied by expert panels from different authorities, such as JECFA, EFSA and FDA, agreeing that they are safe and establishing an ADI for their use as a food additive [32,33]. Furthermore, these final products would entirely be appropriate under the WHO recommendations of reducing sugar intake to 10% of the total daily energy need due to requiring a minimum amount to exert an equivalent sweetness as provided by the sucrose, in addition to being considered low-calorie compounds [34].

## 5. Conclusions

Our findings suggest a comprehensive methodology to use CGTases to modify the chemical structure by maximizing the formation of glucosylated products, consequently improving the flavor of steviol glycosides. The novelty lies in the study of different ranges of key variables and their interaction effects on the transglucosylation process carried out by the CGTases through a response surface approach, providing different evaluation criteria according to the desired response. Additionally, the use of steviol glycosides extracts and the single glycoside rebaudioside A have not been yet explored in the literature to such an extent as reported here. Furthermore, CGTase from three different bacteriological sources were compared, unlike previously in other articles, which supposes a useful tool to be adapted to any other experiment purpose.

By the structural and sensorial analysis, transglucosylation occurred probably in C-13 and/or C-19 sites. These optimal reactions revealed the longer glycosidic chain, obtained by transglucosylation reaction, described until now, consisting of up to 11 glucose units for steviol aglycone of both modified samples. These chemical modifications were carried out with a CGTase from *Geobacillus* sp. and led to an improvement in the quality of taste, more specifically in modified SVglys by significantly reducing the bitter and metallic attributes, consequently resulting in more suitable sweetener substitutes for artificial (synthetic) and caloric sweeteners in food formulations.

## Figures and Tables

**Table 1:** Experimental design by using a fractional factorial design 2^(6-2)^ and the corresponding responses per enzyme and substrate.

	Factors						Responses					
	1	2	3	4	5	6	Synthesized Glucosylated SVglys (mg/mL)			Synthesized Glucosylated RebA (mg/mL)		
Run	Maltodextrin (mg/mL)	Unmodified SVglys or RebA (mg/mL)	Enzyme Activity (U/g acceptor)	Temp (°C)	Time (h)	pH	CGTase *Geobacillus* sp.	CGTase *Paenibacillus macerans*	CGTase *Thermoanaerobacter* sp.	CGTase *Geobacillus* sp.	CGTase *Paenibacillus macerans*	CGTase *Thermoanaerobacter* sp.
1	5	50	5	70	6	5	0.83	0.66	0.80	1.85	0.06	2.49
2	5	5	5	70	1	7	0.92	0.22	1.50	2.69	0.00	2.04
3	5	50	25	50	1	5	1.07	0.55	1.25	2.78	0.21	2.92
4	5	50	5	50	6	7	9.65	7.85	9.83	28.51	4.07	28.49
5	50	5	5	50	6	5	0.84	0.59	1.13	1.94	0.47	2.28
6	5	50	25	70	1	7	3.10	1.21	4.26	8.97	1.83	12.16
7	50	50	25	70	6	7	0.98	0.77	0.76	2.23	0.48	2.04
8	50	5	5	70	6	7	3.34	8.70	4.49	3.01	0.00	12.16
9	50	50	25	50	6	5	6.19	4.01	9.65	26.04	3.71	25.79
10	50	50	5	50	1	7	8.70	2.12	10.86	22.24	0.41	24.98
11	5	5	25	50	6	7	2.07	2.23	4.02	6.81	1.87	10.32
12	50	5	25	70	1	5	1.55	0.73	0.98	2.68	0.00	3.16
13	5	5	5	50	1	5	3.96	2.96	4.47	11.20	1.83	11.24
14	50	5	25	50	1	7	1.10	0.17	1.02	1.33	0.00	2.92
15	5	5	25	70	6	5	11.33	3.73	11.06	27.71	1.54	26.45
16	50	50	5	70	1	5	1.32	1.09	1.48	2.75	0.00	3.12

**Table 2:** Criteria for the optimization obtained from model Equations (1) and (2) by maximizing the response.

Response	Theoretical Result ^a^	Experimental Result ^bc^	Confidence Interval ^d^	
			(-)	( + )
Synthesized glucosylated SVglys (mg/mL)	15.8	17.3 ± 1.0 (6.0%)	12.5	19.1
Synthesized glucosylated RebA (mg/mL)	26.2	26.1 ± 2.4 (9.3%)	22.1	30.3

^a^ *Obtained from model prediction at the optimal settings.* ^b^ *Obtained from an average of additional four runs conducted at the optimal settings.* ^c^ *Standard deviations and relative standard deviations (n = 4) of experimental results are also represented.* ^d^ *Lower (-) and upper (+) confidence interval values calculated to a confidence level of 95%.*

**Table 3:** Sucrose equivalent and potency for unmodified and modified SVglys and RebA by CGTase from *Geobacillus* sp. * standard deviation (*n* = 3).

	Sucrose Equivalent (%)	Potency
SVglys	4.5 ± 2.8 *	140 ± 86
Modified SVglys	4.5 ± 2.5	140 ± 78
RebA	5.3 ± 2.7	223 ± 112
Modified RebA	4.7 ± 2.5	194 ± 106

## Supplementary Materials

The following are available online at https://www.mdpi.com/2304-8158/9/12/1753/s1, Figure S1: Extracted ion chromatograms for glucosylated products of SVglys (black line) and RebA (red line) corresponding to *m/z* 965.4 (a), 1127.5 (b), 1289.5 (c) and 1451.6 (d).
