# Supplementary material for: Transglycosylation of Steviol Glycosides and Rebaudioside A: Synthesis Optimization, Structural Analysis and Sensory Profiles
Source: Foods. 2020 Nov 26;9(12):1753. doi: 10.3390/foods9121753 (PMC7759977; doi:10.3390/foods9121753)
Supplement: Supplementary file 1 [file foods-09-01753-s001.pdf]

Supplementary Material

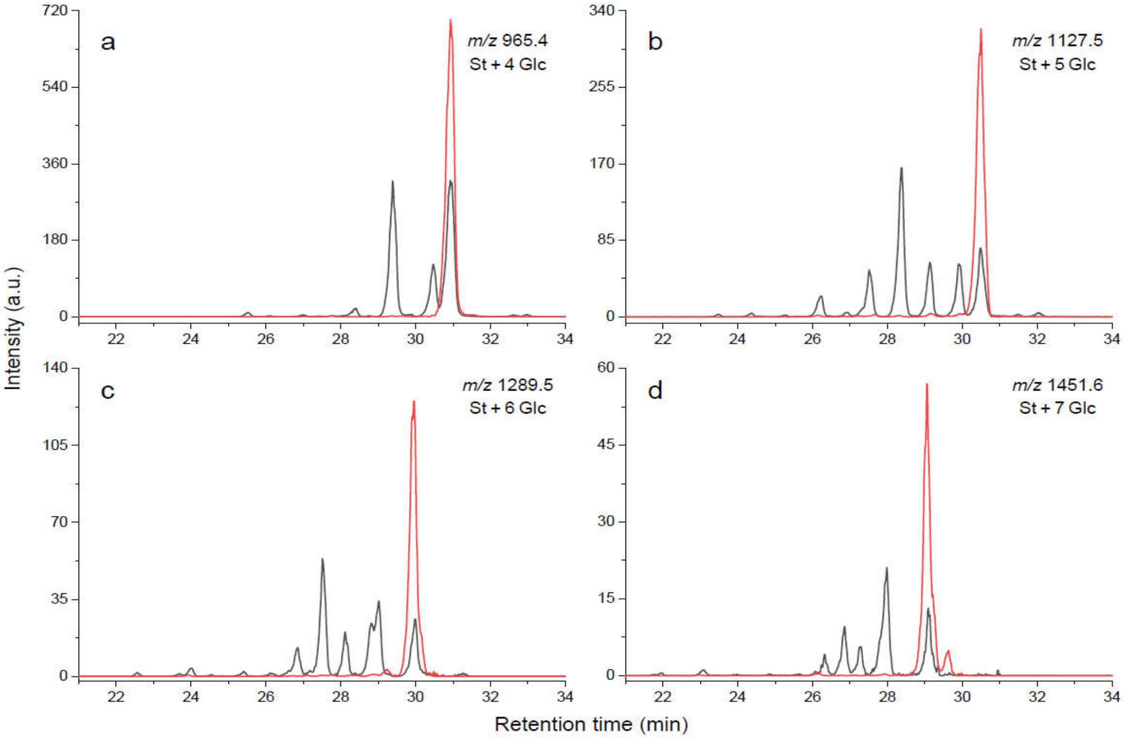

Figure 1. Extracted ion chromatograms for glucosylated products of SVglys (black line) and RebA (red line) corresponding to *m/z* 965.4 (**a**), 1127.5 (**b**), 1289.5 (**c**) and 1451.6 (**d**).
